# Enriching Data Science and Health Care Education: Application and Impact of Synthetic Data Sets Through the Health Gym Project

**DOI:** 10.2196/51388

**Published:** 2024-01-16

**Authors:** Nicholas I-Hsien Kuo, Oscar Perez-Concha, Mark Hanly, Emmanuel Mnatzaganian, Brandon Hao, Marcus Di Sipio, Guolin Yu, Jash Vanjara, Ivy Cerelia Valerie, Juliana de Oliveira Costa, Timothy Churches, Sanja Lujic, Jo Hegarty, Louisa Jorm, Sebastiano Barbieri

**Affiliations:** 1 Centre for Big Data Research in Health The University of New South Wales Sydney Australia; 2 The University of New South Wales Sydney Australia; 3 Medicines Intelligence Research Program School of Population Health The University of New South Wales Sydney Australia; 4 School of Clinical Medicine University of New South Wales Sydney Australia; 5 Ingham Institute of Applied Medical Research Liverpool Sydney Australia; 6 Sydney Local Health District Sydney Australia

**Keywords:** medical education, generative model, generative adversarial networks, privacy, antiretroviral therapy (ART), human immunodeficiency virus (HIV), data science, educational purposes, accessibility, data privacy, data sets, sepsis, hypotension, HIV, science education, health care AI

## Abstract

Large-scale medical data sets are vital for hands-on education in health data science but are often inaccessible due to privacy concerns. Addressing this gap, we developed the Health Gym project, a free and open-source platform designed to generate synthetic health data sets applicable to various areas of data science education, including machine learning, data visualization, and traditional statistical models. Initially, we generated 3 synthetic data sets for sepsis, acute hypotension, and antiretroviral therapy for HIV infection. This paper discusses the educational applications of Health Gym’s synthetic data sets. We illustrate this through their use in postgraduate health data science courses delivered by the University of New South Wales, Australia, and a Datathon event, involving academics, students, clinicians, and local health district professionals. We also include adaptable worked examples using our synthetic data sets, designed to enrich hands-on tutorial and workshop experiences. Although we highlight the potential of these data sets in advancing data science education and health care artificial intelligence, we also emphasize the need for continued research into the inherent limitations of synthetic data.

## Introduction

Clinical data gathered from health care institutions are crucial for enhancing health care quality [1-3]. These data sets can feed into artificial intelligence (AI) and machine learning (ML) models to refine patient prognosis [4,5], diagnosis [6,7], and treatment optimization [8]. Furthermore, statistical models applied to these data sets can uncover association and causal paths [9]. However, stringent privacy regulations protecting patient confidentiality often hamper the prompt availability of these data sets for research and educational usage [10-14].

Gaining access to clinical and health care data sets is a critical aspect of health data science education. This exposure provides trainees with invaluable practical experience, offering profound insights into the complexities of real-world health care scenarios [15]. However, obtaining access to these sensitive data sets is a challenging endeavor—often involving a lengthy process of securing ethics approvals, institutional support, and data clearance [16]. Moreover, the approved users may be required to work on-site under the direct supervision of the data custodian to prevent data leakage [17]. These rigorous security measures, while essential for patient confidentiality, can hamper scalable training of future health data scientists.

During this era of big data, with a soaring demand for skilled health data scientists [18,19], synthetic data sets can bridge the gap between analytical skills and health context comprehension. As Kolaczyk et al [20] astutely asserted, “Theory informs principle, and principle informs practice; practice, in turn, informs theory.”

A promising solution to the lack of clinical and health care data is the utilization of generative AI to generate synthetic data sets. These data sets provide controlled, context-specific learning experiences that parallel real-world situations while maintaining patient privacy. The Health Gym project exemplifies this approach [21]. Leveraging generative adversarial networks (GANs) [22-24], Health Gym creates synthetic medical data sets, establishing a secure yet realistic platform for trainees to hone their health data analytical skills. The data sets, covering key health conditions such as sepsis, acute hypotension, and antiretroviral therapy (ART) for HIV infection, can be accessed at [25]. The project’s open-source code is also available on GitHub at [26] under the MIT License [27].

As an integral part of the Master of Science in Health Data Science Program at the University of New South Wales (UNSW), Australia [28] and a Datathon event [29], the Health Gym synthetic data sets have proven their versatility and effectiveness in enriching health care education. They are freely accessible to the wider research and education community while complying with stringent security standards such as those specified by Health Canada [30] and the European Medicines Agency [31], thus minimizing patient data disclosure risks.

In this viewpoint paper, we discuss the application of Health Gym synthetic data sets, their role in health data science education, and their potential in nurturing proficient health data scientists. We provide adaptable worked examples (accessible through Section A in Multimedia Appendix 1) by using our synthetic data sets, crafted to enrich hands-on tutorial and workshop experiences. We underline the importance of acknowledging the limitations of synthetic data to ensure their valid use in the creation of statistical models and AI applications in health care and the enhancement of health care education. Although synthetic data sets cannot supersede real-world data, they are a vital tool for training future health data scientists and supporting data-driven innovative approaches in health care.

### Ethics Approval

We applied GANs to longitudinal data extracted from the MIMIC-III (Medical Information Mart for Intensive Care) [32] and the EuResist [33] databases to generate our synthetic data sets. This study was approved by the UNSW’s human research ethics committee (application HC210661). For patients in MIMIC-III, requirement for individual consent was waived because the project did not impact clinical care and all protected health information was deidentified [32]. For people in the EuResist integrated database, all data providers obtained informed consent for the execution of retrospective studies and inclusion in merged cohorts [34].

### Health Gym

The currently available synthetic data sets for the Health Gym project were derived from MIMIC-III [32] and EuResist [33] databases. MIMIC-III is a comprehensive database of anonymized health data associated with patients admitted to the critical care units of the Beth Israel Deaconess Medical Center, including data on laboratory tests, procedures, and medications. The EuResist network aims to develop a decision support system to optimize ART for individuals living with HIV, leveraging extensive clinical and virological data.

**Figure 1:**
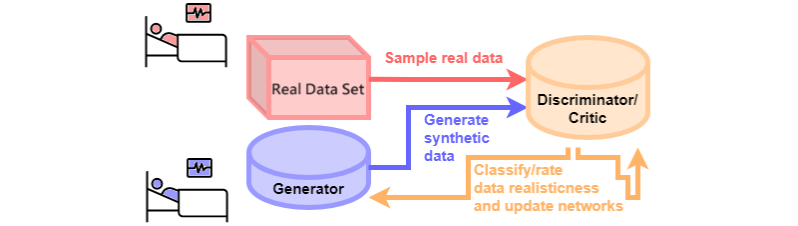
Generative adversarial network setup.

After applying published selection or exclusion criteria, we extracted relevant data from databases that could facilitate the development of patient care algorithms. These data sets, focusing on sepsis, acute hypotension, and ART for HIV, served as the basis for our synthetic data creation. The synthetic data generation employed in the Health Gym was accomplished using GANs. The GAN model, as shown in Figure 1, consists of 2 primary components: a generator and a discriminator. The process starts by sampling real patient records (depicted in pink) and employing the generator to create synthetic patient records (depicted in violet). Both the real and synthetic records are then forwarded to the discriminator network, which is tasked with differentiating the genuine data from the counterfeit. Both networks are trained in an adversarial process—the generator is updated to create more realistic records, while the discriminator is refined to identify generated records more accurately. As a result, the quality of the synthetic data is progressively enhanced, and the synthetic patient records become increasingly representative of the ground truth. The iterative training concludes when the discriminator can no longer reliably distinguish the synthetic records from the real records. Refer to more details in Kuo et al [21].

Leveraging generative AI, Health Gym provides highly authentic clinical data sets, enriching health care education. Each data set undergoes rigorous quality assessment and security verification (detailed in Section B of Multimedia Appendix 1). These synthetic data sets foster engaging learning experiences, aiding educators in developing tailored educational strategies. The following sections will illuminate the application of Health Gym in university-level courses, exemplified through ART for HIV data set.

### Synthetic ART for HIV Data Set

The Health Gym data sets contain mixed-type longitudinal data, including numerical, binary, and categorical variables. They encompass patient demographics, vital signs measurements, and pathology results. The data sets hence reflect the complexities of real-life data, thereby making them suitable for training health data scientists in university courses. This paper will primarily delve into the application of synthetic data in health care education focusing on the ART for HIV data set. Readers interested in the sepsis and the acute hypotension data sets should refer to Section C in Multimedia Appendix 1.

#### Data Set Description

Our synthetic HIV data set, informed by the selection or exclusion criteria proposed by Parbhoo et al [35] and drawn from the EuResist database, targets individuals living with HIV who initiated therapy after 2015 per the World Health Organization’s guidelines [36]. ART for HIV typically includes a mix of 3 or more antiretroviral agents from at least 2 distinct medication classes. The dynamism of ART lies in its frequent regimen modifications resulting from various circumstances such as treatment failure due to poor adherence or viral resistance, intolerance to ART, clinical events such as pregnancy or coinfections, or optimization of therapy to support better adherence, reduce drug-drug interactions, maximize ART response, or prevent the emergence of drug-resistant viral strains [36,37].

In addition to ART information, the data set encompasses vital indicators of ART success and disease progression, namely, viral load (VL) and CD4 cell count. Successful ART is often indicated by VL below 1000 copies/mL, while a CD4 cell count exceeding 500 cells/mm^3^ signifies healthy immunological status [36]. The complex interactions of these elements in our data set create a rich learning platform for health data science education.

Table 1 encapsulates the data set’s 3 numeric, 5 binary, and 5 categorical variables. Numeric variables include VL, CD4 cell count, and relative CD4 laboratory test results. Treatment regimens follow those of Tang et al [38], breaking down the ART regimen into several parts. The data set includes 50 combinations of 21 unique medications. The antiretroviral medication classes are nucleoside/nucleotide reverse transcriptase inhibitors (NRTIs), nonnucleoside reverse transcriptase inhibitors (NNRTIs), integrase inhibitors (INIs), protease inhibitors (PIs), and pharmacokinetic enhancers (pk-En). We deconstructed the ART regimen into its constituent parts: base drug combination (base drug combo), complimentary INIs (comp INIs), comp NNRTIs, extra PIs, and extra pk-En. The base drug combo primarily consists of NRTIs, with inclusion of other antiretroviral classes as well.

Recognizing the notable amount of missing data in the original EuResist database, we added a suffix (M) to variables to denote whether measurements were recorded at specific time points. In the authentic data set, measurements were reported at 24.27% (129,835/534,960) for VL (measured), 22.21% (118,815/534,960) for CD4 (measured), and 85.13% (455,411/534,960) for drug (measured). The absence of some CD4 and VL records may be attributable to specific clinical practices and the frequency of test requests [39-42]. For instance, it is common for clinicians to discontinue requesting a CD4 cell count if the previous result exceeded 500 cells/mm^3^ and the individual had an undetectable VL. Similarly, VL is typically measured in the first 3 months, at 6 months, 12 months, and then annually.

**Figure 2:**
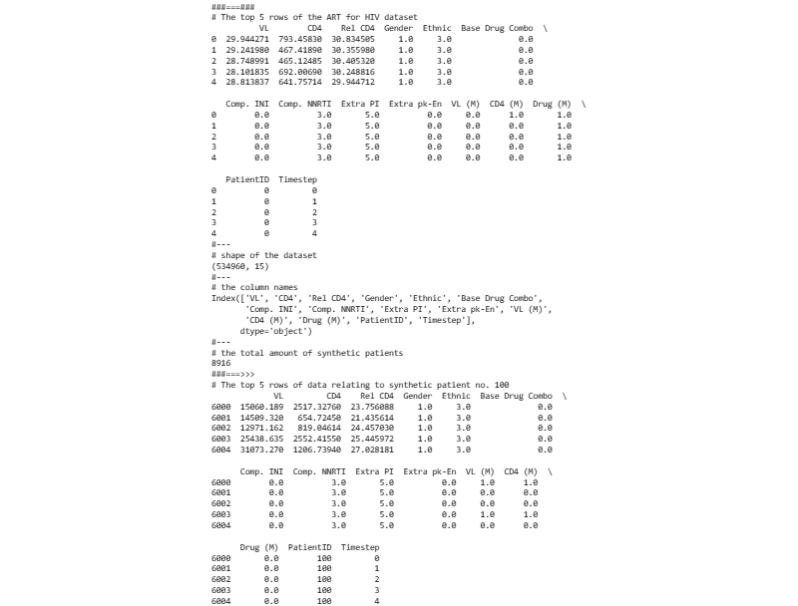
Inspecting the antiretroviral therapy for an HIV data set (output of the code in Figure 3).

**Figure 3:**
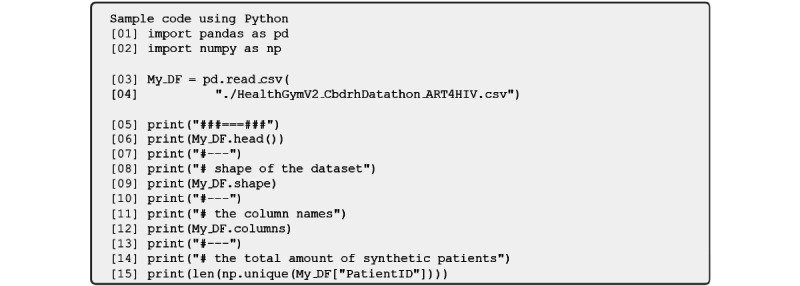
Code in Python for generating the output shown in Figure 2. This code uses pandas [44] and NumPy [45]. Base drug combo: base drug combination; comp INI: complementary integrase inhibitor; comp NNRTI: complementary nonnucleoside reverse transcriptase inhibitor; PI: protease inhibitor; pk-En: pharmacokinetic enhancer; VL: viral load.

Constructed using the GAN model developed by Kuo et al [43], this data set comprises 8916 synthetic patients tracked over 60 months, resulting in 534,960 records (8916 *×* 60). Figure 2 showcases a sample generated by the code in Figure 3 [44,45]. Each record features 15 columns, including a patient identifier, a time point, and 13 ARTs for HIV variables highlighted in Table 1. The synthetic data sets can be freely accessed in [46] and [47] on Figshare, a digital platform for research output sharing.

**Table 1:** The variables of antiretroviral therapy in the HIV data set.

Variable name	Data type	Unit	Valid categorical options
Viral load (VL)	numeric	copies/mL	N/A^a^
Absolute count for CD4 (CD4)	numeric	cells/µL	N/A
Relative count for CD4 (Rel CD4)	numeric	cells/µL	N/A
Gender	binary	N/A	Male, Female
Ethnicity (Ethnic)	categorical	N/A	Asian, African, Caucasian, other
Base drug combination (Base drug combo)	categorical	N/A	FTC^b^ + TDF^c^, 3TC^d^ + ABC^e^, FTC + TAF^f^, DRV^g^ + FTC + TDF, FTC + RTVB^h^ + TDF, other
Complementary integrase inhibitor (Comp INI)	categorical	N/A	DTG^i^, RAL^j^, EVG^k^, not applied
Complementary nonnucleoside reverse transcriptase inhibitor (Comp NNRTI)	categorical	N/A	NVP^l^, EFV^m^, RPV^n^, not applied
Extra protease inhibitor (Extra PI)	categorical	N/A	DRV, RTVB, LPV^o^, RTV^p^, ATV^q^, not applied
Extra pharmacokinetic enhancer (Extra pk-En)	binary	N/A	False, True
Viral load measured (VL) (M)^r^	binary	N/A	False, True
CD4 (M)	binary	N/A	False, True
Drug recorded (M)	binary	N/A	False, True

^a^N/A: not applicable.

^b^FTC: emtricitabine.

^c^TDF: tenofovir disoproxil fumarate.

^d^3TC: lamivudine.

^e^ABC: abacavir.

^f^TAF: tenofovir alafenamide.

^g^DRV: darunavir.

^h^RTVB: ritonavir.

^i^DTG: dolutegravir.

^j^RAL: raltegravir.

^k^EVG: elvitegravir.

^l^NVP: nevirapine.

^m^EFV: efavirenz.

^n^RPV: rilpivirine.

^o^LPV: lopinavir.

^p^RTV: ritonavir.

^q^ATV: atazanavir.

^r^(M): measured.

#### Applications and Case Studies

This section highlights the use of our synthetic ART for HIV data set in a collaborative Datathon event and as an effective teaching tool at UNSW for medical education.

### Center for Big Data Research in Health Data Science Datathon

The synthetic data set for ART for HIV was a central component of the UNSW Center for Big Data Research in Health Datathon [48], an event merging theoretical learning with practical application. The Datathon was an enriching exercise in multidisciplinary collaboration. The event involved 6 teams, with a total of 24 participants, offering a tangible experience in data analysis. The student teams were supported by a group of mentors—a blend of data scientists, clinicians, health professionals, and government health informatics specialists from a local health district in Sydney, Australia [49]. The data scientists and the panel of authors of the Health Gym project (ie, Kuo et al [21]) elaborated on the technical aspects and navigated the participants through the intricacies of data analysis, including the assumptions we made to use the data (eg, time 0 corresponded to the date of ART initiation, the laboratory tests occurred before modifications in therapy). Meanwhile, clinicians and health professionals provided their expertise to guide students toward meaningful research questions (eg, discussing VL and CD4 count monitoring, drug-drug interactions, and metabolic toxicity [50]). Government health informaticians, experienced in electronic medical records and real-world population health application and impact, evaluated the usefulness of the students’ findings.

This collaborative effort facilitated a comprehensive learning experience, encompassing the development of analytical models, data visualization, and effective communication of research outcomes. Using our synthetic data sets, participants gained valuable insights into working with data sets that emulate real-world health scenarios, thereby providing a bridge between theoretical academia and practical execution.

We summarize the findings of the 2 participating teams below. Detailed reports for Team 1 and Team 2 can be found in Section D and Section E of Multimedia Appendix 1, respectively. In addition, the associated codes for the 2 teams can be found in Section A of Multimedia Appendix 1.

#### Findings of Team 1

Team 1 investigated the effectiveness of medications, categorized by antiretroviral class, in achieving HIV suppression. Utilizing survival analysis, they assessed the time between the initiation of ART to the first occurrence of viral suppression, defined as VL below 1000 copies/mL [36]. They also assessed the time to CD4 cell count exceeding 500 cells/mm^3^ [51], which indicates a healthy immunological status.

With Cox proportional hazards models [52] featuring time-varying covariates, the team identified particular antiretroviral agents associated with viral suppression. These findings were purely associative due to data set limitations, which did not account for factors such as age, socioeconomic status, comorbidities, and concurrent medications (of other illnesses).

#### Findings of Team 2

Team 2 focused on predicting the necessity of altering an individual’s ART regimen over a 5-year time span, factoring in disease flare-ups, resistance, or side effects. They formulated a “sliding search” function that generated individual records for each 12-month period, with predictions for antiretroviral modification and adherence to therapy in the subsequent year by using neural networks. The team’s methodology produced promising results, with an accuracy rate of 78% in predicting antiretroviral modification and 93% in predicting adherence to therapy. The algorithm detected trends in CD4 and VL results across the 12-month periods, which appeared to be the key predictive features. In addition, the team suggested that there could be potential benefits from exploring recurrent neural networks (eg, long short-term memory [53]).

### Serving as UNSW Coursework Materials

Beyond their utility in the Datathon, our synthetic data sets contribute to UNSW courses in the Master of Science in Health Data Science Program [54], namely, HDAT9800 Visualization & Communication and HDAT9510 Machine Learning II.

HDAT9800 teaches future health data scientists the skills to visually communicate complex data effectively to diverse audiences. The course emphasizes the significance of clear data visualization and advocates for transparency and reproducibility in scientific work. It employs R [55] and Python [56] to demonstrate best practices in data analysis and visualization. Our synthetic data sets provide rich resources to enhance the learning in this setting. For instance, Marchesi et al [57] used our data sets to present patient states via t-distributed stochastic neighbor embedding visualization techniques [58].

Meanwhile, HDAT9510 explores advanced modern ML algorithms and methods such as convolutional neural networks [59], autoencoders [60], and reinforcement learning (RL) [61]. As the synthetic data sets consist of time-series variables, students can develop both feedforward and recurrent neural networks. See example models built using our data set in Marchesi et al [57] with recurrent neural networks and even decision trees [62] and hidden Markov models [63], as in a similar data set suggested by Wu et al [64]. Furthermore, with the presence of nonnumeric variables, students can learn about embedding [65]—transforming nonnumeric levels into real-valued vectors so that similar levels that are closer in the vector space carry more analogous meaning. The presence of missing data in the synthetic data sets also encourages students to formulate plausible assumptions about the structure of the clinical data set prior to data modelling.

We provide 3 adaptable worked examples using our ART for HIV data set, suitable for workshops and lectures. The associated codes for the worked examples can be found in Section A of Multimedia Appendix 1. Our synthetic data set supports a variety of student engagements, from understanding complex data structures to developing advanced RL algorithms for optimizing clinical interventions. Moreover, the low patient disclosure risk associated with our data sets (refer to Section B in Multimedia Appendix 1) eliminates the need for ethics approval [66]. This makes these data sets ideal for a range of settings—from small seminars to larger lecture groups.

#### Worked Example 1

**Figure 4:**
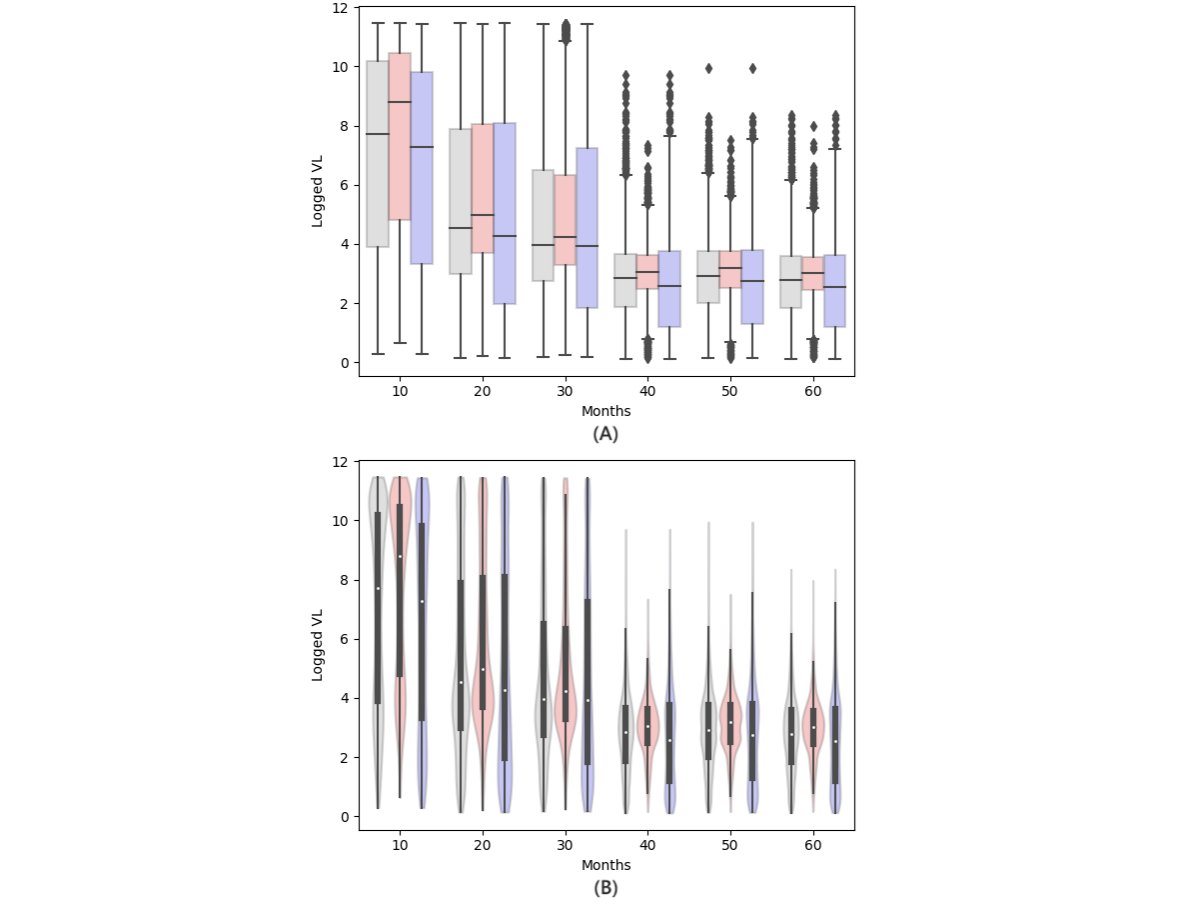
Viral load distribution. Subplot (A) shows a box plot comparison of viral load across base drug combinations across time, and subplot (B) shows a violin plot comparison of viral load across base drug combinations across time. Grey indicates all patients, red indicates those initiating treatment with FTC + TDF (emtricitabine + tenofovir disoproxil fumarate), and blue indicates those initiating treatment with 3TC + ABC (lamivudine + abacavir). VL: viral load.

The first exercise, focused on data visualization using Python, compares VL trends over time among patients who commenced their ART with different base drug combos, against the general trend in all patients. The results of our worked example are depicted in Figure 4.

This multifaceted exercise requires students to create sub–data sets based on specific starting base drug combos (ie, FTC + TDF [emtricitabine + tenofovir disoproxil fumarate] and 3TC + ABC [lamivudine + abacavir]), extract data for defined periods, and familiarize themselves with box and violin plots [67]. They are also tasked with organizing the visual data as side-by-side plots.

Through this exercise, students will understand the limitations of box plots, which cannot visualize underlying data distributions. They will learn about the additional insights provided by advanced plotting techniques such as violin plots. In addition, students will note that people who start with FTC + TDF and those who start with 3TC + ABC display similar patterns as the overall ART for HIV cohort. The overlap of the interquartile ranges across all box plots indicates a consistent behavior.

#### Worked Example 2

**Figure 5:**
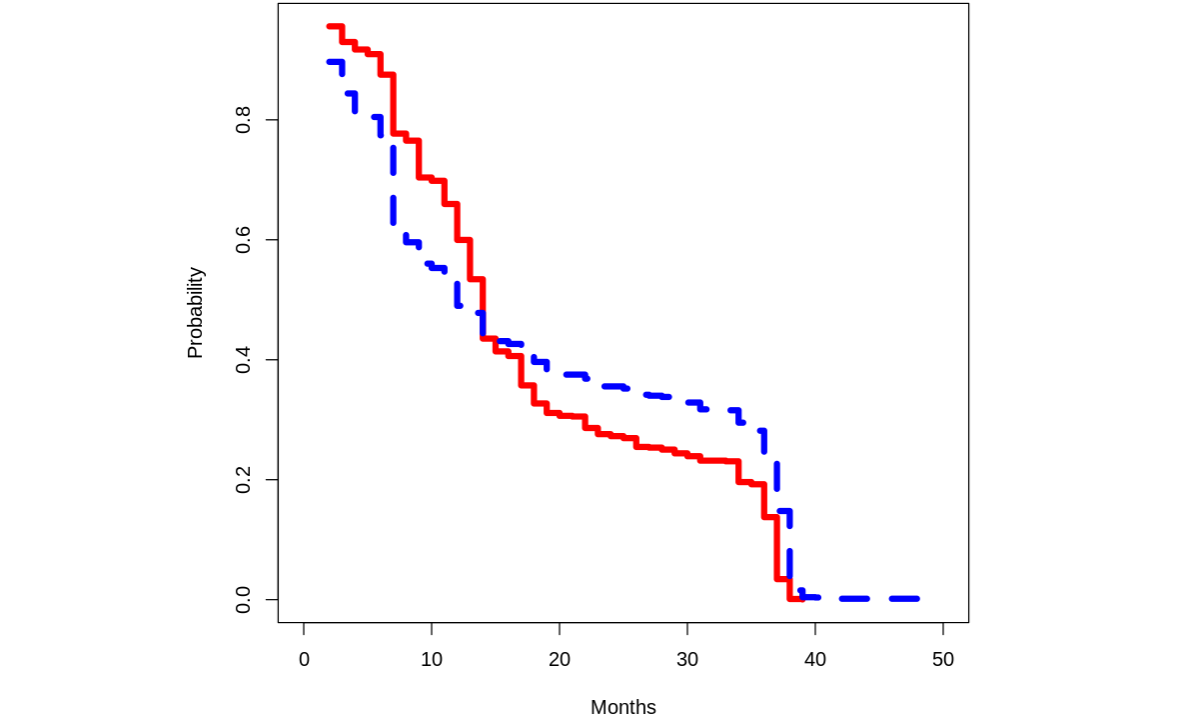
Time-to-event estimation of viral load suppression for viral load lower than 1000 copies/mL. Red indicates those initiating treatment with FTC + TDF (emtricitabine + tenofovir disoproxil fumarate) and blue for those initiating treatment with 3TC + ABC (lamivudine + abacavir).

The second exercise delves into survival analysis using R [55], building on insights from the initial data visualization task. The exercise continues to compare results among people starting with the base drug combo of FTC + TDF and those initiating with the base drug combo of 3TC + ABC. The goal is to estimate the time necessary for a person on ART to successfully suppress their VL. The results of our worked example are depicted in Figure 5.

This task proves to be more complex than the first, requiring HIV domain knowledge, such as an understanding that a reasonable threshold for ART in HIV treatment is 1000 copies/mL [36]. This threshold indicates slowed viral replication and immune system damage. Thus, students should select patients who commence ART with VL above 1000 copies/mL (ie, not experiencing the outcome of interest at baseline).

Creating an appropriate data set for survival analysis is key, as is pinpointing when each patient’s VL first drops to or below 1000 copies/mL. In addition, students need to grasp the concept of right censoring [68] and utilize Kaplan-Meier curves [69] for time-to-event estimations. This offers an opportunity to engage with the influential survival package [70] in the R language. Upon examining the results in Figure 5, students will note no significant differences in the timing of VL suppression between people who started with the base drug combo of FTC + TDF and those who initiated with the base drug combo of 3TC + ABC.

#### Worked Example 3

The third exercise immerses students in the process of developing an RL agent using Python. RL is a type of ML that learns an evidence-based policy to connect states (the current scenario) to actions (the potential responses to that scenario). In the context of our HIV treatment example, states refer to the representation of the patient’s current health status and medication history, while action refers to the selection of medication to use in response to each state.

**Figure 6:**
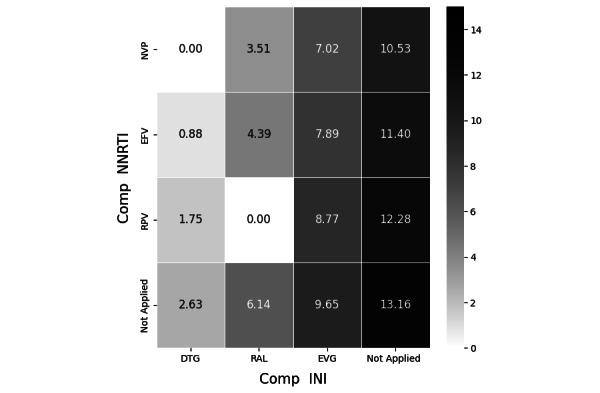
Visualizing the learned reinforcement learning policy. Comp INI: complementary integrase inhibitor; Comp NNRTI: complementary nonnucleoside reverse transcriptase inhibitor; DTG: dolutegravir; EFV: efavirenz; EVG: elvitegravir; NVP: nevirapine; RAL: raltegravir; RPV: rilpivirine.

The RL agent selects an action based on a policy that optimizes for maximum cumulative rewards, even as environments evolve. This approach has particular relevance to health care. Clinicians often need to adapt treatment plans to each patient’s unique circumstances, and RL can help them to individualize treatment durations, dosages, or types. For example, they may alter the regimen, class, or specific agents of medication to better serve the patient’s needs. The outcomes of our example are visualized in Figure 6. This exercise highlights the potential of RL to enhance patient care through personalization—an aspect that is becoming increasingly important in today’s medical landscape.

This complex exercise is designed for advanced students, posing challenges across multiple dimensions. It commences with data wrangling, where students scrutinize numeric variable distributions and evaluate the necessity for transformations such as rescaling, normalization [71], power transformation [72], or Box-Cox transformation [73].

In the next stage, students encounter categorical feature representation for medication regimens, practicing their skills in implementing embeddings. Advanced students can explore transfer learning for feature representation [74]. This exercise also presents real-world challenges, requiring students to handle mixed-type data progression. During the model fitting phase, students must employ suitable ML models, distinguishing between RL method archetypes [75] and considering their clinical implications.

Data visualization is the next task, encouraging students to articulate model-derived insights into digestible visuals for a diverse audience. The concluding phase involves refining assumptions and model performance, incorporating multiple tests to identify optimal hyperparameters [76]. Here, students peek into the “black box” nature of ML and gain an intuition for effective module combinations [77-79]. This step becomes critical for causal inference tasks that necessitate rigorous input data validation [80].

Figure 6 showcases the strategy employed by an RL agent in HIV therapy. Heatmaps visualize the relative frequencies of chosen actions (ie, the selected antiretroviral), where each tile represents a unique action and its frequency as a proportion of all actions. The example output shows that the RL agent consistently suggests the EFV + RAL (efavirenz + raltegravir)—a combination of comp NNRTIs and comp INIs—4.39% of the time, while never recommending the RPV + RAL (rilpivirine + raltegravir) combination. More information on the steps taken to create the output for this task can be found in Section F of Multimedia Appendix 1.

## Discussion

This paper demonstrates the transformative potential of synthetic health data sets in health care education, especially in the evolving context of generative AI integration. These data sets provide a realistic representation of real-world health data complexities while preserving patient confidentiality, facilitating experiential learning, skills enhancement, and interdisciplinary collaboration. However, this significant stride toward AI integration in education is not without challenges, and the creation of AI models trained on curated quality data sets emerges as a promising research area.

Despite our best efforts, the Health Gym synthetic data sets might not fully capture the complexity and diversity of real-world scenarios. For instance, some critical health determinants such as socioeconomic status [81] and comorbidities [82] are missing from the ART for HIV synthetic data sets. The absence of these factors mirrors the broader issues concerning data accessibility [83], particularly when it involves specialized or rare disease information. Furthermore, synthetic data might overlook uncontrolled variables or confounders inherent in real-world data [84,85], posing pedagogical challenges. However, this limitation is not solely attributable to our methodology. Since the socioeconomic status variable is not present in the EuResist database, our model lacked the necessary reference data from the outset.

In the field of health data science, proficient data set management and curation are essential due to the decentralized nature of health care data collection. Many entities contribute to health data, each using their own systems [86]. Privacy laws such as Australia’s Privacy Act 1988 [87] and the United States’ Health Insurance Portability and Accountability Act [88] complicate the sharing of data, resulting in a fragmented view of patient information.

Record linkage techniques [89] such as probabilistic matching [90] bridge this gap by linking disparate data records, offering a more comprehensive view of a patient’s health. Nevertheless, our synthetic data sets, despite their potential, carry limitations such as the absence of a master linkage key [91], thereby reducing their applicability in university courses for data management and curation. Having such linked data sets are also great for health data science students to test hypotheses on the effects of comorbidities. Our experiences from the Datathon suggest that the Health Gym synthetic data sets are best used for creating algorithms to enhance patient care within specific disease management paradigms.

Our Health Gym initiative leverages a unique application of generative AI, differing from those used in emerging AI-assisted chatbots, which have also shown promise as potent educational tools. AI chatbots, with their personalized and interactive responses using large language models, can significantly incite interest and foster self-directed learning in medical students [92]. However, advanced AI tools such as OpenAI’s ChatGPT [93] and Google’s BARD [94] bring with them valid concerns around precision, reliability, potential misuse, and adherence to academic integrity [95,96]. In contrast, the synthetic clinical data sets, the generative product of our Health Gym project, offer controlled, scenario-specific learning environments that closely reflect real-world conditions while preserving patient privacy.

Access to clinical data sets is integral to health data science education, but the necessity of maintaining patient confidentiality can hinder the training of future health data scientists on a larger scale. This may exacerbate the digital divide [97,98], which is a prominent challenge in the broader AI integration into education. As we shift toward AI-driven educational resources, it is essential to prioritize equitable access across varied socioeconomic backgrounds. Future research should evaluate the long-term effects of AI on student learning, clinical judgment, patient outcomes, and the development of educational resources for effective AI integration. The secure, realistic synthetic data sets of Health Gym may provide a valuable solution, potentially facilitating equal access to educational materials.

### Conclusion

Despite their limitations, the Health Gym synthetic health data sets have demonstrated their value in educating and training future health data scientists. Their integration into interdisciplinary platforms such as Datathon illustrates their potential in promoting collaborative learning, skills enhancement, and innovative research. In addition, synthetic data sets offer a learning platform that balances realistic health scenario representation with data privacy preservation.

Although we have primarily demonstrated the utility of Health Gym’s synthetic data sets by using the ART for HIV data set, we emphasize the importance of the additional acute hypotension and sepsis data sets that we have developed (see Section C in Multimedia Appendix 1). These data sets broaden the scope of medical education by providing insight into managing illnesses in intensive care units, encompassing a unique set of measurements and pathology information. As such, these synthetic data sets offer students an enriched, realistic learning environment for health data science education, complementing the HIV data set and furthering the applicability and versatility of synthetic health data.

The majority of generative ML research is centered on computer vision [99,100] and, to a lesser extent, natural language processing [101], leaving clinical health care data relatively unexplored. This gap suggests a valuable opportunity for future research, particularly considering that clinical data being longitudinal, mixed-type time series variables have a fundamentally different nature. As demonstrated in our prior studies [21,43,102], we have ascertained that our synthetic data sets attain a robust level of validity and are readily available to support both clinical research and medical pedagogy; predictive models instantiated on our synthetic data sets parallel those of the original data sets in their characteristics. We will focus our future work on comparing synthetic data sets created using various generative ML architectures, for example, GANs, variational autoencoders [103], diffusion probabilistic models [102,104], and transformer-based models [105].

GANs, like other ML models, can only optimize according to predefined optimization functions. Given the current lack of research on the use of GANs in health care, more utility studies are necessary to fully comprehend the potential of our synthetic data sets. We are committed to continuing collaboration with clinicians and health professionals to better understand the practical strengths and weaknesses of synthetic data sets, including how to better evaluate and contain the risk of private information disclosure. Through these collective efforts, we aim to improve the quality of synthetic data sets, enhancing hands-on learning experiences for students in health data analytics.

## Appendix

Multimedia Appendix 1 Supplementary data.

## Abbreviations

3TC: lamivudine
ABC: abacavir
AI: artificial intelligence
ART: antiretroviral therapy
Base drug combo: base drug combination
Comp INI: complementary integrase inhibitor
EFV: efavirenz
FTC: emtricitabine
GAN: generative adversarial network
INI: integrase inhibitor
MIMIC: Medical Information Mart for Intensive Care
ML: machine learning
NNRTI: nonnucleoside reverse transcriptase inhibitor
NRTI: nucleotide reverse transcriptase
PI: protease inhibitor
pk-En: pharmacokinetic enhancer
RAL: raltegravir
RL: reinforcement learning
RPV: rilpivirine
TDF: tenofovir disoproxil fumarate
UNSW: University of New South Wales
VL: viral load
