# Supplementary material for: Enriching Data Science and Health Care Education: Application and Impact of Synthetic Data Sets Through the Health Gym Project
Source: JMIR Med Educ. 2024 Jan 16;10:e51388. doi: 10.2196/51388 (PMC10828942; doi:10.2196/51388)
Supplement: Multimedia Appendix 1 [file mededu_v10i1e51388_app1.docx]

# Supplementary Materials for the paper

Enriching Data Science and Healthcare Education:

Application and Impact of Synthetic Datasets through the Health Gym Project

**Nicholas I-Hsien Kuo**∗*,*1, **Oscar Perez-Concha**∗*,*1, **Mark Hanly**1

**Emmanuel Mnatzaganian**2, **Brandon Hao**2, **Marcus Di Sipio**2, **Guolin Yu**2, **Jash Vanjara**2, **Ivy Cerelia Valerie**2,

**Juliana de Oliveira Costa**3, **Tim Churches**1, **Sanja Lujic**1, **Jo Hegarty**4, **Louisa Jorm**1, **Sebastiano Barbieri**1

^∗^Equal contribution.

^1^Centre for Big Data Research in Health, the University of New South Wales, Sydney, Australia

^2^the University of New South Wales, Sydney, Australia

^3^Medicines Intelligence Research Program, School of Population Health, the University of New South Wales, Sydney, Australia

^4^Sydney Local Health District, Sydney, Australia

Corresponding author: Nicholas I-Hsien Kuo (n.kuo@unsw.edu.au)

In our study, we delve into the utilisation of synthetic datasets from our Health Gym project (Kuo et al., 2022), which were generated using generative adversarial networks (GANs) (Goodfellow et al., 2014). Given the shortage of publicly accessible clinical data, these synthetic datasets provide a valuable resource for medical education. For clarity and brevity in the main manuscript, we have relegated numerous useful discussions to this appendix to complement the main document. Here, readers can access a wealth of additional information, including the access to associated codes suit- able for university-level tutorials and workshops, a discussion on our data quality assessment and security verification, as well as detailed outlines and reports of our experimental setups.

# Content of Tables:

§A: Associated Codes and Repositories

§B: Quality Assessment and Security Verification

§C: The Sepsis Dataset and the Acute Hypotension Dataset

§D: An In-Depth Report of Team 1

§E: An In-Depth Report of Team 2

§F: Visualising the Policy Learnt by the RL Agent

# Associated Codes and Repositories

In this appendix, we provide a collection of useful codes and repositories readily adaptable for university-level tutorials and workshops.

You can find the codes for generating the synthetic datasets on our GitHub repository:

<https://github.com/Nic5472K/ScientificData2021_HealthGym>

The datasets from our project can be downloaded from the Health Gym website:

<https://healthgym.ai/>

Alternatively, the sepsis and acute hypotension datasets can be accessed from Physionet: [https://physionet.org/content/synthetic-mimic-iii-health-gym/1.0.](https://physionet.org/content/synthetic-mimic-iii-health-gym/1.0.0/) [0/](https://physionet.org/content/synthetic-mimic-iii-health-gym/1.0.0/)

The ART for HIV datasets are available on Figshare:

<https://figshare.com/authors/Nicholas_Kuo/12369910>.

**Datathon Reports**

Access Team 1’s analysis code through the following link:

<https://github.com/Emm-mnatz/DATATHON_TEAM_1>

An in-depth discussion of their report can be found in Section D of the Appendix.

Team 2’s analysis code is available at:

<https://github.com/jashvanjara/UNSW-Datathon-2023/>

A detailed discussion of their report is presented in Section E of the Appendix.

**Worked Examples Adaptable for University-Level Courses**

Firstly, the worked example for data visualisation is accessible via: [https://colab.research.google.com/drive/1lwgNxlxxXq012aqcG_](https://colab.research.google.com/drive/1lwgNxlxxXq012aqcG_VfBfaXFIoRMuMb?usp=sharing) [VfBfaXFIoRMuMb?usp=sharing](https://colab.research.google.com/drive/1lwgNxlxxXq012aqcG_VfBfaXFIoRMuMb?usp=sharing).

Secondly, the worked example for survival analysis can be found at: [https://colab.research.google.com/drive/12dDi9x-Ts-ilIjlLF35Y1_](https://colab.research.google.com/drive/12dDi9x-Ts-ilIjlLF35Y1_KUjZwYoUUK?usp=sharing) [KUjZwYoUUK?usp=sharing](https://colab.research.google.com/drive/12dDi9x-Ts-ilIjlLF35Y1_KUjZwYoUUK?usp=sharing).

Finally, the worked example for Reinforcement Learning (RL) is available here: [https://colab.research.google.com/drive/1trUH6EY7h8M53lIGYXF4LKq709zzI8F5?](https://colab.research.google.com/drive/1trUH6EY7h8M53lIGYXF4LKq709zzI8F5?usp=sharing) [usp=sharing](https://colab.research.google.com/drive/1trUH6EY7h8M53lIGYXF4LKq709zzI8F5?usp=sharing).

# Quality Assessment and Security Verification

Our synthetic datasets need to be both realistic and secure to advance downstream machine learning algorithms and enrich medical and health data science education. We assessed the verisimilitude of our synthetic datasets by conducting a multi-layered quality check. Comparing the synthetic variable distributions with their real counterparts, deploying a series of statistical hypothesis tests (*i.e.,* the Kolmogorov–Smirnov test (Hodges, 1958), t-test (Yuen, 1974), F-test (Snedecor & Cochran, 1989), and the three-sigma-rule test (Pukelsheim, 1994)), and affirming inter-variable correlations.

Moreover, we validated the practical utility of these synthetic datasets. We found that RL agents trained on our synthetic datasets recommended actions closely aligning with those suggested by RL agents trained on the real datasets. See the alignment results for sepsis and for acute hypotension in Kuo et al. (2022); and see the alignment results for the ART in HIV in Kuo et al. (2023a). This assures us that synthetic data-driven insights mirror those we might gain from their real-world counterparts.

While realism is crucial, we must safeguard real patient privacy in order to make our synthetic data accessible to all. There were no patient-identifying variables in the acute hypotension dataset, but there were demographics (*e.g.,* age, gender, and ethnicity) in the sepsis and the ART for HIV datasets. A verification process was hence required to ensure that a known target’s sensitive real- world information could not be inferred from our synthetic data.

We first ensured that no real records could be found in our synthetic datasets simulated using GANs. Subsequently, we ascertained that the risk of a successful *population-to-sample attack* (El Emam et al., 2020) is negligible, falling well below the 9% threshold set by Health Canada (2014) and the European Medicines Agency (2014) – as stated in Kuo et al. (2022), a successful synthetic-to-real attack was estimated to be 0.80% for the sepsis dataset. Also see Kuo et al. (2023a), a successful synthetic-to-real attack was estimated to be 0.095% for the ART for HIV dataset.

# The Sepsis Dataset and the Acute Hypotension Dataset

The synthetic datasets we developed for acute hypotension and sepsis are grounded in the MIMIC- III database and follow selection criteria respectively defined in Gottesman et al. (2019) and Ko- morowski et al. (2018).

The synthetic acute hypotension dataset includes 20 clinical variables, observed hourly over a 48- hour span for each patient. It includes data on blood pressures, specific lab results, mechanical ventilation parameters, Glasgow Coma Scale score, and details on fluid boluses, and vasopressors, to name a few. It also tracks data missingness, a key aspect in healthcare, suggesting the potential need for specific lab tests (Sharafoddini et al., 2019).

The synthetic sepsis dataset includes 44 variables, including vital signs, lab results, mechanical ven- tilation data, and patient-specific measurements. This dataset has 80 hours of hospital stay records per patient, and it is presented in 4-hour windows. Some parameters, like the PaO2/FiO2 ratio, shock index, and SOFA score, are intentionally left out as they can be calculated from other variables.

For open access to these synthetic datasets, visit PhysioNet, a freely available medical research data repository managed by the MIT Laboratory for Computational Physiology, as detailed in Kuo et al. (2023c). For additional information on these datasets, refer to Kuo et al. (2022).

# An In-Depth Report of Team 1

- 1. Datathon Research Problem:

The participants engaged in HIV treatment, investigating which medications, classified by antiretro- viral class, were most effective in achieving HIV viral suppression. This problem was framed within two primary parameters: tracking the first instance of viral load dropping below 1,000 copies/mL and observing the first instance of CD4 cell count rising above 500 cells/mm^3^.

- 1. Datathon Research Approach:

The participants initiated the process with data preparation, regrouping variables for more suitable utilisation. Specifically, the participants found that the medications of DRV and RTVB may be duplicated in both the Base Drug Combo and Extra PI variables. The research was carried out within the context of a survival analysis framework, where medications’ effectiveness was evaluated based on the duration until a patient reached specific clinical endpoints.

- 1. Techniques Used:

After preparing the data, the participants implemented survival analysis techniques, utilising Cox Proportional Hazards models with time-varying covariates. These models were grouped by patients’ IDs to reflect the dynamic nature of the covariates over time and across individuals.

- 1. Research Findings:

From the analysis, the students identified specific antiretroviral drugs linked to achieving viral sup- pression. However, these findings were associations and not causations due to the dataset’s limita- tions. The analysis accounted for variables like patients’ gender, ethnicity, and base CD4 levels, but relevant factors like age, socioeconomic status, comorbidities, and concurrent medications couldn’t be controlled for (as these variables were missing from the synthetic ART for HIV dataset), thus constraining the comprehensiveness and applicability of the results.

- 1. Pros and Cons of the Current Synthetic Dataset:

The synthetic dataset used during the Datathon offered a rich platform for exploring real-world research questions and conducting hands-on data manipulation. It provided an opportunity to under- stand practical complexities involved in data analysis. However, the participants encountered chal- lenges with the dataset, including inconsistencies in recording certain medications (see Research Approach) and the absence of certain significant variables like age and socioeconomic status (see Research Findings). Despite these limitations, the synthetic dataset was still considered an effective and beneficial tool in understanding and solving real-world problems.

# An In-Depth Report of Team 2

- 1. Datathon Research Problem:

The team focused on predicting the need for changes in drug combination in HIV patients over time. The motivation for this research problem arose from a blend of factors such as the dataset provided, clinical experience, understanding of HIV, and lessons learned from orientation lectures. Alternate research goal included predicting drug adherence.

- 1. Datathon Research Approach:

The team initially identified that drug combinations often needed adjustments over time, forming the basis of the chosen research question. The approach revolved around a unique “sliding search” strat- egy, considering 12-month periods and predicting outcomes for the subsequent year. This method- ology was intended to capture temporal trends more accurately.

- 1. Techniques Used:

Data pre-processing involved creating a function that generated individual rows for each 12-month period, with a corresponding label indicating whether the drug combination changed and the pa- tient’s compliance over the next year. The team implemented a two-layer neural network to compare variables across each 12-month period.

- 1. Research Findings:

The implemented neural network yielded an accuracy of 78% for predicting drug combination changes and 93% for predicting patient compliance. Further insights were gained by analysing in- put weights, particularly with CD4 and viral counts. It was found that these variables had a cyclical pattern, suggesting that the algorithm was identifying trends in these levels to predict outcomes.

- 1. Pros and Cons of the Current Synthetic Dataset:

The Datathon presented a unique opportunity to explore medical and informatics theories while gaining hands-on experience in data preparation, model building, and evaluation. However, the experience also highlighted some challenges. Time constraints limited the ability to apply more complex models, such as recurrent neural networks, which could have potentially yielded better results. In terms of performance metrics, the limited time also meant that evaluation was primarily done using accuracy. In an ideal scenario, the model’s performance would have been assessed using additional metrics like confusion matrices for more comprehensive understanding of model strengths and weaknesses.

# Visualising the Policy Learnt by the RL Agent

We train our RL agents using observational variables *D_O_* and action variables *D_A_* from our synthetic ART for HIV dataset in Table 1. In our experiments, *D_O_* includes the three numeric variables – VL, CD4, and Rel CD4 – at the current time step *t* and all medications used at the previous time step *t −* 1. The three numeric variables provide the patient’s health state, and information from previous regimens is factored in to consider potential drug-resistant viral strains.

For the worked example, we use Comp. INI and Comp. NNRTI to span the action space *A*, resulting in 16 (= 4*×*4) unique actions. Following Liu et al. (2021), we define clinical states from observation variables *D_O_*. Cross decomposition (Wegelin, 2000) is first applied to reduce *D_O_* dimensionality to 5. Then, we label each *D_O_* data point using their associated clusters by performing K-Means clustering (Vassilvitskii & Arthur, 2006) with 100 clusters.

Our RL method is batch-constrained Q-learning (Fujimoto et al., 2019). For each setup, the policy is updated for 50 iterations with a step size of 0.01. The RL policy is updated using the reward function from Parbhoo et al. (2017). The reward function (reward*_t_*) is defined as:

*−*0*.*7 logVL*_t_* + 0*.*6 logCD4*_t_,* if VL*_t_* is above detection limits, and

5 + 0*.*6 logVL*_t_* if VL*_t_* is below detection limits*.*

(1)

We note that while we use cells/*µL* as the unit for CD4*_t_* count in Table 1, Parbhoo et al. use cells/mL for the reward computation in Equation (1).
